# Validating the Chinese Version of the Personal Accountability Measure for Assessing Teachers’ Perceptions and Experiences of Teacher Accountability in China

**DOI:** 10.3390/bs13020145

**Published:** 2023-02-09

**Authors:** Kwok Kuen Tsang, Wanying Zhang, Yuan Teng, Huan Song

**Affiliations:** 1 Department of Education Policy and Leadership, The Education University of Hong Kong, Hong Kong 999077, China; 2 Faculty of Education, Beijing Normal University, Beijing 100875, China; 3 Faculty of Education, Central China Normal University, Wuhan 430079, China; 4 Center for Teacher Education Research, Beijing Normal University, Beijing 100875, China; 5 Institute of Plateau Science and Sustainable Development, Qinghai Normal University, Xining 810008, China

**Keywords:** teacher accountability, felt accountability, accountability disposition, validation

## Abstract

The study aims to validate the Chinese version of Personal Accountability Measure (PAM-Ch), which is used to assess the subjective aspect of teacher accountability, by surveying 1146 teachers enrolled in professional development courses offered by a public university in Beijing. The validation process involved two phases. First, the samples were randomly divided into three subgroups—for subgroup 1 (*n* = 390), exploratory factor analysis was computed; for subgroup 2 (*n* = 359), confirmatory factor analysis (CFA) was computed; and, for subgroup 3 (*n* = 381), a new CFA was performed for cross-validation. Second, Cronbach’s α, composite reliability (CR), average variance extracted (AVE), maximum shared variance (MSV), and average shared variance (ASV) were calculated for testing the reliability and validity. Throughout the process, three measurement models were tested for the adaptation of the PAM-Ch in this study. The results found that Model 2 was the best fit for the data, whose factor loadings ranged from 0.72–0.95 for internal accountability (factor 1) and 0.75–0.89 for external accountability (factor 2). The CRs of these two factors were 0.963 and 0.916, respectively, and the AVE values were 0.790 and 0.645, respectively, indicating that the PAM-Ch is a reliable and valid measure.

## 1. Introduction

Teachers are regarded as the gatekeepers of education since they play a critical role in nurturing students with proper knowledge, skills, attitudes, and values for the sustainable development of society. Nevertheless, school organization is traditionally a loosely coupled system that has insufficient mechanisms and measures to monitor teachers’ work for quality of education [1]. Thus, there is no doubt that some teachers may teach poorly or perform inadequately in the job [2], leading to an ineffective and inefficient school system [3]. To improve the situation, since the 1980s, governments across the globe have initiated educational accountability reforms to hold teachers accountable and responsible for their decisions, actions, and outcomes and keep them responsive to stakeholders, such as the state authorities, students, and parents [4,5,6,7,8]. Numerous studies suggest that the reforms, despite their good intentions, have deleterious effects on teachers’ wellbeing and quality of teaching because accountability measures, such as performance indicators, parent choice, and school inspection, are inclined to degrade, deskill, and intensify teachers’ work, leading teachers to feel deprofessionalized, demoralized, stressed, anxious, and burnt out [9,10,11,12,13,14,15]. However, the major limitation of these studies is that they only regard accountability as an objective institutional environment affecting all teachers’ work and wellbeing in the same way but neglect the dispositions of accountability subjectively experienced by teachers [16]. When the subjective aspect of accountability is taken into account, as Erdağ [17] indicated, researchers find that the perceptions and feelings toward the same accountability environment vary between teachers with different characteristics, such as gender, school grade, and subject taught. In other words, even though teachers are situated in the same accountability environment, their perceptions and experiences of it may differ [18]. Thus, disregarding the subjective aspect of teacher accountability may restrict our understanding of teacher accountability and its impacts on teachers [18,19].

To enable researchers to measure the subjective aspect of teacher accountability, Rosenblatt [16] developed the Personal Accountability Measure (PAM). According to Erdağ [18], PAM is the only instrument that explicitly addresses the subjective aspect of teacher accountability. Nevertheless, PAM is a new measure that has only been validated in a few countries, such as Israel [16] and Turkey [18]. Therefore, it is important to validate PAM in other regions to advance our understanding of teacher accountability in different cultural contexts and of its relationship to teachers’ work and wellbeing in different parts of the world.

The present study aims to validate PAM in mainland China, where great emphasis has been placed on teacher accountability. For instance, teachers have been socially expected and pressured to be accountable and responsible for students’ merits measured by standardized tests, such as the Gaokao, the university entrance examination [20]. Moreover, since 1983, the government has developed a teacher accountability system consisting of a variety of measures, such as educational inspection, teacher performance evaluation, and merit pay, to monitor teachers’ work [5]. Recently, the government has proposed deepening the teacher accountability system to effectively and systemically evaluate and monitor teachers’ quality and professionalism [21]. As a result, testing the factor structure of PAM in mainland China will enhance our knowledge of Chinese teacher accountability and in turn generate recommendations for Chinese education policy makers to improve their accountability system for the sustainable development of teachers’ work and wellbeing. Moreover, as Rosenblatt and Wubbels [8] noted, teachers’ perceptions and experiences of accountability may be affected by societal cultures. In other words, it is important for researchers to conduct comparative studies to learn about the cultural differences in teacher accountability. To achieve this goal, a standardized and validated measure is needed. Thus, validating PAM in mainland China will not only extend the measure to Chinese contexts, but also facilitate future comparative studies. Accordingly, the study would prefer to answer the following research question: Is the PAM a reliable and valid measure in mainland China?

## 2. Teacher Accountability

In general, accountability means that someone is held accountable by others [22,23]. In school settings, teacher accountability is regarded as a social mechanism and a virtue that pressures teachers to explain and justify their decisions and actions [24]. As a social mechanism, teacher accountability refers to formal systems and measures, such as teacher appraisal, performance indicators, internal audit, and parent choice, which are used to evaluate and judge teachers’ conduct in teaching by stakeholders, namely, government, school leaders, parents, and students, leading to rewards or sanctions [25]. Additionally, teacher accountability as a virtue is about professional norms, values, and ethics requiring teachers to reflectively monitor themselves in certain ways [23]. Teacher accountability as a social mechanism emphasizes the external expectations and demands of stakeholders through formal accountability systems and measures whether teacher accountability as a virtue is in accordance with those expectations and demands from professional norms, values, and ethics, leading to self-monitoring with little external security [19]. Rosenblatt [16] refers to the former as external accountability and the latter as internal accountability.

At the organizational level, both external and internal accountability can be regarded as the dispositions of an institutional environment explicitly and implicitly requiring teachers to give an account of their decisions, actions, and outcomes to stakeholders [8]. At the personal or individual level, teacher accountability functions only when teachers perceive the dispositions of accountability and agree to be accountable based on expectations and demands [8,25,26]. Therefore, researchers, e.g., [8,16,17,19,25,26,27] suggest that it is necessary to pay attention to the subjective aspect of accountability if we want to have an in-depth analysis of the effects of accountability on teachers.

To conceptually distinguish from the objective aspect of teacher accountability, researchers refer to the subjective aspect of teacher accountability as accountability dispositions [8,16], felt accountability [25,28], individual-level accountability [29], or personal accountability [17]. In the literature, these terms are interchangeable and used to suggest accountability as a subjectively perceived or experienced phenomenon [8,27]. For example, Orakcı, Dilekli, and Erdağ [19] define felt accountability “as the perception that the employee will be evaluated by someone else and asked for an explanation for his or her actions and decisions when necessary”. Similarly, Hall and Ferris [27] suggest that individual-level accountability is the perceived expectation about one’s performance and outcomes that will be evaluated by others and that rewards and punishments are contingent on the expected evaluation. Moreover, according to Rosenblatt and Wubbels [8], accountability disposition is a state of mind, i.e., a subjective interpretation of a structured accountability context.

Accordingly, teacher accountability refers to teachers’ perceived responsibility and answerability regarding their decisions, actions, and outcomes [16]. It consists of two dimensions, namely, internal and external accountability. The former is teachers’ accountability dispositions regarding external demands and expectations of others through formal mechanisms and measures, while the latter is teachers’ feelings of duty and commitment to competence and development based on their professional norms, values, and ethics [18].

## 3. PAM for Teachers

There are a few well established measures used to assess teachers’ accountability dispositions in the literature. Although measures, such as the Teacher Accountability Questionnaire, developed by Rahmatollahi and Zenouzagh [30], and the Accountability Scale for Quality Education, developed by Bandele and Ajayi [31], assess teacher sense of accountability toward students, parents, school, society, and professional community of teachers with good reliability and validity, they do not distinguish the external and internal expectations and demands upon the teachers. Therefore, these measures may not facilitate researchers to investigate the structure of teacher accountability disposition comprehensively. To overcome the limitation, Rosenblatt [16] develops PAM to assess the personal disposition of teachers to act accountably at work. The PAM consists of 13 items measuring two factors of teacher accountability, namely, external accountability (six items) and internal accountability (seven items). Each item is rated on a 5-point Likert-type scale ranging from 1 (strongly disagree) to 5 (strongly agree). PAM was first validated by a series of studies conducted on Israeli teachers [16]. These studies indicate that Cronbach’s α for the whole PAM and its dimensions, i.e., external accountability and internal accountability, are 0.83, 0.71, and 0.85 in Israel, respectively. Moreover, the studies establish convergent validity with regard to concrete accountability scenarios in teachers’ work life, emerging from focus group interviews, construct validity with regard to a spectrum of personal characteristics conceptually similar to accountability, such as goal orientation, work ethic, and conscientiousness assessed by the instruments developed by Vandewalle [32], Miller, et al. [33], and Goldberg [34], respectively, and predictive validity in regard to teacher work absence.

Recently, Erdağ [18] conducted a study to validate PAM in Turkey. By surveying 643 Turkish teachers, he confirmed the two-factor model of PAM by both exploratory factor analysis and confirmatory factor analysis. According to the study, Cronbach’s α and composite reliability (CR) for external accountability are 0.93 and 0.67, respectively, and Cronbach’s α and CR for internal accountability are 0.67 and 0.67, respectively. His further analysis shows that PAM achieves convergent and discriminant validity in the Turkish sample. Different from the original PAM [16], Erdağ [18] indicates that the Turkish version of the PAM only contains 12 items instead of 13 items—five items for external accountability and seven items for internal accountability. He explains that the difference in item number may be related to cultural differences between Turkey and Israel, leading to different perceptions and experiences of accountability between teachers in the two societies. Moreover, in another study, Erdağ [17] tested the invariance of the Turkish version of the PAM across gender, tenure, teaching branch, and school grade by multigroup confirmatory factor analysis. The findings suggest that the two-factor structure of PAM is consistent across the subgroups of teachers. According to CR coefficients indicated in the study, the internal consistency is adequate among the subgroups for internal accountability (0.794 < CR < 0.963) and external accountability (0.577 < CR < 0.758).

Similarly, the study conducted by Orakcı, Dilekli, and Erdağ [19] confirmed the two-factor structure of the PAM in Turkey. They found that CR coefficients for external accountability and internal accountability are 0.58 and 0.84, respectively, implying that the subscales of PAM are reliable. Moreover, the study also shows that external accountability and internal accountability are significantly correlated with teachers’ innovative thinking and responsible teaching.

## 4. Materials and Methods

### 4.1. Participants and Procedure

The study surveyed teachers who enrolled in professional development courses from April to June 2020 offered by a public university in Beijing, China, and they completed an online questionnaire. The online questionnaire was created with Questionnaire Star, an online platform for questionnaire surveys. This platform was used because it only displayed the IP address, rather than other personal identifiers of participants. In addition, the questionnaire did not require participants to give their names and other identifiable information. Accordingly, the online questionnaire survey should be able to keep participants anonymous [35].

Every teacher enrolled in the professional development courses received the link to the questionnaire and was invited to complete the online questionnaire at the last session of the courses. To ensure the participants’ rights, the first page of the online questionnaire introduced the purpose of the study, clarified the participants’ role in the study, ensured no negative consequences if they decided to terminate the survey at any time, and elaborated on how the collected information would remain confidential and be used for research purposes only. After reading the information, they could click the start button if they consented to participate in the survey study.

A total of 1306 questionnaires were collected from teachers in mainland China. In order to ensure the validity and authenticity of the data, we eliminated questionnaires with a missing rate of more than 50% and a duplication rate of more than 90%. Finally, 1146 valid questionnaires were obtained, and the effective rate was 87.749%. These included language teachers (31%), mathematics teachers (16.3%), English teachers (11.7%), biology teachers (4.8%), political science teachers (3.6%), ideology teachers (6.0%), PE teachers (8.1%), and other teachers (18.5%). The sample covered three school levels: elementary, middle, and high school, accounting for 49.4%, 18.4%, and 32.2% of the total sample, respectively. Among them, 75% were female teachers. The majority came from Anhui (23.56%), followed by Shaanxi (17.71%), Guangdong (10.65%), Guangxi (9.16%), Jilin (8.16%), Shandong (6.02%), Henan (4.62%), Hubei (3.58%), Beijing (3.23%), Inner Mongolia (2.97%), Jiangxi (1.83%), and other provinces or cities (8.55%), including Hebei, Fujian, Shanghai, Tianjin, Heilogjiang, Liaoning, Shanxi, Yunnan, Guizhou, Jiangsu, Sichuan, Zhejiang, Nanjing, Xinjiang, and Chongqing.

### 4.2. Measures

#### Personal Accountability Measure (PAM)

First, the original PAM developed by Rosenblatt [16] was translated from English to Chinese by a member of the research team. Second, 30 in-service teachers in Beijing were invited to review the Chinese items and provide feedback. Based on the feedback, the language was polished to enhance readability and make the items better fit the Chinese context. Third, another research team member translated all items back into English. Finally, three members of the research team compared the back translations with the original PAM and, in turn, finalized the Chinese version of the PAM (PAM-Ch). Similar to the original PAM, the PAM-Ch was designed to assess two dimensions of teacher accountability: external accountability (6 items) and internal accountability (7 items). All items were rated on a 5-point Likert-type scale ranging from 1 (strongly disagree) to 5 (strongly agree).

### 4.3. Data Analysis

For statistical processing, we used SPSS 26.0 for exploratory factor analysis (EFA) and probability–probability plot (PP Plot). Amos 26.0 was used for confirmatory factor analysis (CFA).

The validation process for the PAM-Ch involved two separate phases. The first phase was designed to investigate the validity of the scale and its cross-validation, whereas the second aimed to examine internal consistency and reliability, as well as convergent and discriminant validity.

In the first stage, the researchers randomly divided the entire dataset (*n* = 1146) into three subgroups to assess the scale validity of PAM. For subgroup 1, exploratory factor analysis (EFA) was performed (at least 100 cases), and the sample size was 390. For subgroup 2, confirmatory factor analysis (CFA) was performed (at least 150 cases), and the sample size was 359 (CFA1). For Group 3 (n =381), a new validated factor analysis (CFA2) was performed for cross-validation of the optimal factor model from the previous CFA1 sample. Several scholars suggest that such an approach can avoid overfitting and, thus, improve the replicability of the model [18,36,37], which can also further protect the proposed model from confirmation bias [38].

In the second stage, the reliability and validity of the scale were tested with the whole sample (*n* = 1146). In terms of reliability, Cronbach’s α and CR were calculated in this stage; in terms of validity, the average variance extracted (AVE), maximum shared variance (MSV), and average shared variance (ASV) of the scale were calculated in this stage.

## 5. Results

### 5.1. Preliminary Analysis

First, two trained coders entered and cross checked the data for the EFA, CFA1, and CFA2 subgroups together, and no univariate outliers were detected. Second, the probability–probability plot (PP Plot) was used to test whether the samples conformed to a normal distribution. The PP plot is a graph based on the relationship between the cumulative proportion of variables and the cumulative proportion of the specified distribution [39,40]. The PP plot allows researchers to check whether the data conform to the specified distribution. When the data obey the assumed distribution, the points corresponding to each data point are approximately distributed in a straight line at the right diagonal position in the graph [41,42]. The detrended PP plot is a supplement to the PP plot test, and the data can be considered to obey the distribution under test when the distribution of the data under test is around the 0 scale [40].

**Figure 1:**
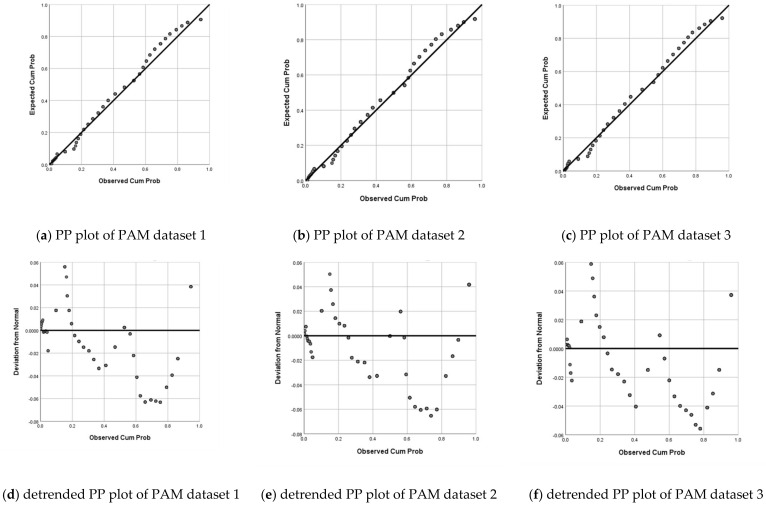
Results of normal distribution test.

The results are shown in Figure 1, and the scatter points of the PP plots of the three subgroups could match the diagonal lines better. Meanwhile, the detrended PP plot with the cumulative probability as the horizontal coordinate and the deviation from the standard normal distribution, as the vertical coordinate, showed that the vertical coordinate units in the three data samples are between −0.07 and 0.06. The deviations of the three data samples were small relative to the cumulative probability1 and could be considered as basically conforming to the normal distribution.

### 5.2. Exploration of the Scale Structure through EFA

Based on the results of the preliminary analysis, the researchers conducted KMO and Bartlett’s test on dataset 1. The results showed that the KMO measure of sampling adequacy was 0.918, which was greater than 0.8. Meanwhile, the approximate chi-square of Bartlett’s test of sphericity was 5327.533, df was 78, and p was 0.000, which passed the 1% significance test. The PAM-Ch data were well suited for factor analysis.

**Figure 2:**
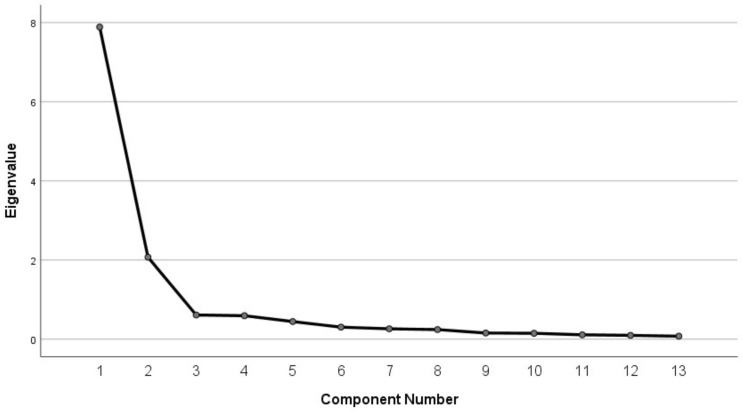
Scree plot.

According to the Scree plot (Figure 2), the fold tended to level off after 3 and drop sharply before that, indicating that it was more appropriate to extract two common factors for the 13 items of the PAM-Ch. Meanwhile, the statistics of principal component extraction for the 13 items of the PAM-Ch showed that there was a total of two factors with initial eigenvalues greater than 1, and the cumulative explained variance was 76.604%, showing that the extraction of two factors was better for the explanation of the original data. Among them, Factor 1 had an eigenvalue of 7.887 and a percentage of explained variance of 44.538%; Factor 2 had an eigenvalue of 2.072 and a percentage of explained variance of 32.066% (see Table 1).

The factor attribution of each topic could be determined based on the rotated component matrix (Table 2). Among them, INT1-INT7 belonged to Factor 1, whose factor loadings were all greater than 0.685 and labelled “internal accountability”; EXT 1–6 belonged to Factor 2, whose factor loadings were all greater than 0.597 and labelled “external accountability”. It is worth noting that the loadings of EXT6 on Factor 1 and Factor 2 were both greater than 0.5, indicating that this item had low discriminant validity.

### 5.3. Evaluation of Measurement Models through CFA

The different measurement models were further tested by validated factor analysis on different subsamples. Based on EFA and the literature on teacher accountability [16], three measurement models were tested for the adaptation of the PAM-Ch. Model 1 was a one-dimensional, one-factor model [43] with 13 question items tested in the CFA1 (*n* = 359) subsample. Model 2 was a first-order, two-dimensional, two-factor model [16], with 13 question items tested in the CFA2 subsample (*n* = 381). Model 3 was a first-order, two-dimensional, two-factor model based on EFA proposed with 12 question items, where EXT6 was removed. Model 3 was tested in the CFA1 (Model 3a) and CFA2 (Model 3b) subsamples.

**Figure 3:**
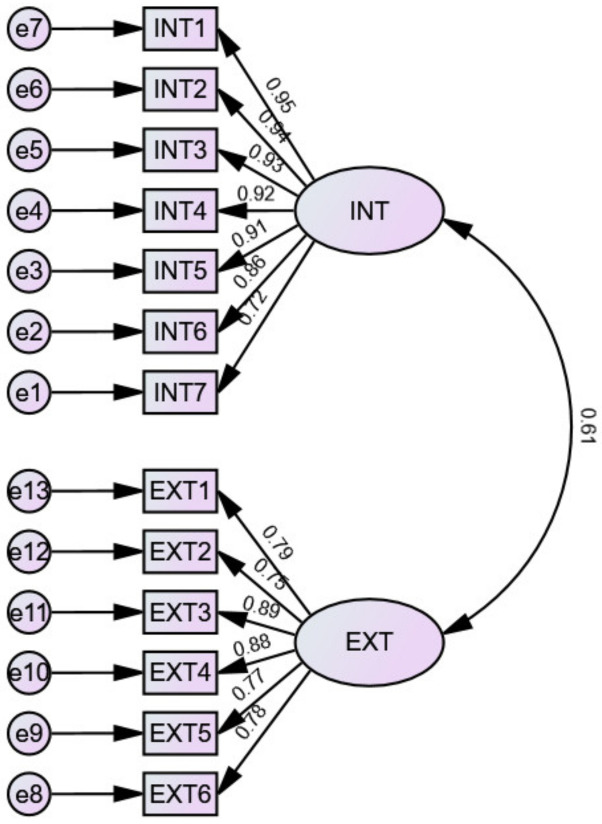
Model 2 path diagram.

**Figure 4:**
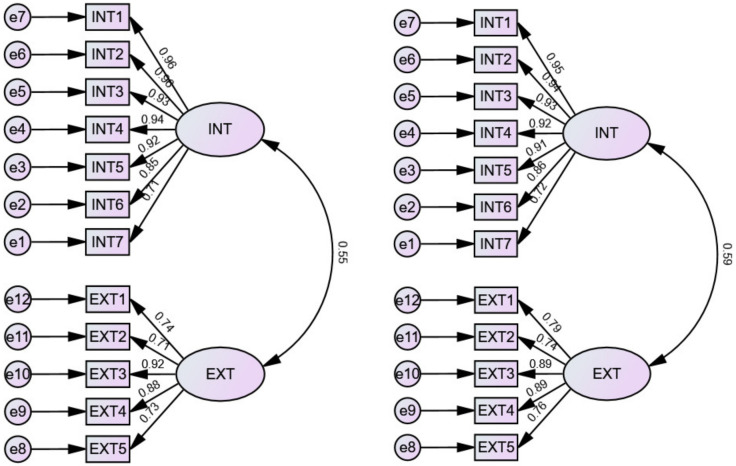
Model 3a and Modal 3b path diagrams.

The results are shown in Table 3, Figure 3 and Figure 4. Model 1 fits the data poorly (χ2/df = 21.124, RMSEA = 0.237, NII = 0.740, RFI = 0.688, IFI = 0.699), indicating that the PAM-Ch was not a one-factor scale. The fit of Model 2 to the data was acceptable (χ2/df = 5.502, RMSEA = 0.109, NII = 0.933, RFI = 0.918, IFI = 0.945), all standardized regression weights in Model 2 were significant (*p* < 0.001), and factor loadings ranged from 0.72–0.95 for Factor 1 (internal accountability) and 0.75–0.89 for Factor 2 (external accountability). The factor correlation was 0.61. In addition, the fit of Model 3a and Model 3b data also reached an acceptable level. However, by comparing the model fit, factor loadings, and interfactor correlations, it could be found that Model 2 was the best fit for the data. Thus, in this study, the researchers decided to use Model 2 for further data analysis.

### 5.4. Internal Consistency Reliability, Convergent and Discriminant Validity

Reliability analysis, i.e., the internal consistency test, was used to test whether the results of the data collected by the questionnaire were consistent. This study used Cronbach’s α coefficient. Normally, Cronbach’s α coefficient of 0.6 or more indicates good consistency in the data results of the questionnaire. In this study, the researchers used the whole sample (*n* = 1146) for reliability testing and found that the Cronbach’s α coefficient of the whole scale of the PAM-Ch was 0.943, which was greater than 0.9, indicating that the overall reliability of the scale was good. The Cronbach’s α coefficient of internal accountability was 0.960, and the Cronbach’s α coefficient of external accountability was 0.911, both of which were greater than 0.9, indicating that the reliability of each dimension was good.

Finally, the researchers examined the convergent and discriminant validity of the PAM-Ch, and the output metrics included AVE and CR values. Typically, AVE values greater than 0.5 and CR values greater than 0.7 indicate good data convergent and discriminant validity. As shown in Table 4, the CRs of the two factors (internal accountability and external accountability) in this study were 0.963 and 0.916, respectively, and the AVE values were 0.790 and 0.645, respectively, indicating that the PAM had good convergent and discriminant validity in the sample of this study.

## 6. Discussion

The aim of the study is to validate the PAM-Ch in mainland China. Similar to the original PAM designed by Rosenblatt [16], the PAM-Ch is composed of 13 items measuring two factors, namely, international accountability (seven items) and external accountability (six items). The findings show that the two-factor with a 13-item model has a better fit to the data than the one-factor with a 13-item model and the two-factor with the 12-item model. Moreover, the findings suggest that the whole PAM-Ch and its two subscales have good internal reliability. Further data analyses illustrate that the PAM-Ch has good composite reliability and good convergent and discriminant validity in the sample of the study.

Although the findings suggest the same factor structure of the PAM-Ch as the original PAM, it is noted that EXT6—“get credit for the success of your classes”—may be convergent with both external accountability and internal accountability. The convergence implies that Chinese teachers experience both external and internal forces holding them accountable and responsible for the success of their classes. Such an experience of teachers may be related to the sociocultural structure of mainland China’s education system. In mainland China, the government has initiated a series of policies aimed at holding teachers accountable for good performance since the 1980s [44]. One of the far-reaching policies is the merit pay system that links teacher salary with their merits in teaching. Based on the principle of “good performance, good pay”, teachers who can help students become successful in schooling will be externally credited, leading to greater financial rewards [45,46]. Moreover, there is a teacher honour system in mainland China that aims at awarding outstanding teachers with honourary titles, such as backbone teacher, excellent teacher, and subject leader as professional recognitions leading to symbolic power to the awardees in the education system [47,48]. Thus, teachers in mainland China may perceive receiving credit for the success of their classes as a result of external accountability measures such as the merit pay system and teacher honour system. On the other hand, Lo, Lai, and Wang [44] observe that a major feature of Chinese teacher professionalism is the emphasis on students. As they note, “to enable students to learn and to perform well academically, to mould them into persons of good character, are considered the major responsibility of teachers” and teacher responsibility “represents their academic and moral obligations towards their students” [44]. Thus, helping students become successful may be a professional norm, value, or ethic regulating teachers’ work in mainland China. When a Chinese teacher demonstrates responsibility, his or her professional identity may be verified [49], and his or her professionalism may also be socially recognized [45]. In other words, teachers may also interpret the EXT6 as a statement about their experience of internal accountability, in addition to external accountability, in mainland China’s education system.

The significance of the present study is twofold. First, it provides a valid and reliable measure to assess the disposition of teacher accountability in mainland China. The measure is important for educational research, since there is no validated measure for education researchers to investigate the impacts of teacher accountability on Chinese teachers’ work and wellbeing. Due to the lack of such a measure, when education researchers investigate the behavioural and psychological issues of teachers, such as commitment, stress, and burnout, in the Chinese context of teacher accountability, they tend to attribute these issues to teacher accountability without any robust test of the relationship between teacher accountability and behavioural and psychological issues, e.g., [45,50,51]. Nevertheless, teacher accountability is not just an objective institutional environment; it also encompasses teachers’ subjective perceptions and experiences of the expectations, pressures, and demands of others upon them [8,16]. In other words, the effects of teacher accountability may vary between teachers with different backgrounds and characteristics because personal backgrounds and characteristics may shape one’s perception and experiences of teacher accountability differently [17]. Therefore, the validated PAM-Ch will enable education researchers to test the impacts of teacher accountability on teachers’ work and wellbeing to develop more robust explanations in further research.

Second, teacher accountability is a worldwide phenomenon, but it does not mean that the effects of teacher accountability on teachers’ work and wellbeing are the same in different societies because teachers with different sociocultural backgrounds may perceive and experience teacher accountability differently [8]. Therefore, if education researchers would prefer to compare the similarities and differences in the relationship between teacher accountability and teachers’ work and wellbeing in different societies, a standardized measure that is validated in a variety of societies is needed. As PAM is the only measure addressing the subjective aspect of teacher accountability [18], it is important to validate it in different societies for the purpose of comparative research. Thus, it is worthwhile for the present study to investigate its validity and reliability in mainland China because it provides the validated PAM-Ch for further research that aims to compare teacher accountability between Chinese societies and other societies.

This study has some limitations. First, although the sample size of the study is 1306 and there are 1146 valid cases, the study only surveyed teachers from professional development courses offered by a university in Beijing. In mainland China, not every teacher can attend such professional development courses if he or she does not have the school administrators’ nomination [52]. The nominated teachers are generally those who have high job commitment, strong potential to become teacher leaders, or good teaching performance perceived by school administrators. In other words, the findings may not represent the whole teacher population in China. Therefore, further studies should repeat the present study by sampling participants who can represent typical teachers across mainland China. Second, even though the study validates the PAM-Ch, it does not guarantee that the PAM-Ch can be applied to all Chinese societies. There are three major Chinese societies, namely, mainland China, Hong Kong, and Taiwan. Although they share the same Chinese culture, their socioeconomic and education systems are different. The differences may affect teachers’ perceptions and experiences of teacher accountability. Therefore, further studies should also validate the PAM-Ch in Hong Kong and Taiwan. Finally, as Erdağ [18] notes, self-report measures may exclude potential social desirability in teacher accountability scores. Therefore, further studies on teacher accountability should include a measure of social desirability to identify any social desirability bias when the participants respond to the questionnaire.

## Figures and Tables

**Table 1:** Total Variance Explained.

Component	Initial Eigenvalues			Extraction Sums of Squared Loadings			Rotation Sums of Squared Loadings		
	Total	% of Variance	Cumulative %	Total	% of Variance	Cumulative %	Total	% of Variance	Cumulative %
1	7.887	60.667	60.667	7.887	60.667	60.667	5.790	44.538	44.538
2	2.072	15.937	76.604	2.072	15.937	76.604	4.169	32.066	76.604
3	0.610	4.693	81.297						
4	0.592	4.557	85.854						
5	0.445	3.421	89.275						
6	0.304	2.338	91.613						
7	0.262	2.012	93.626						
8	0.243	1.866	95.492						
9	0.156	1.197	96.689						
10	0.148	1.142	97.830						
11	0.110	0.849	98.680						
12	0.096	0.740	99.420						
13	0.075	0.580	100.000						

Note. Extraction Method: Principal Component Analysis.

**Table 2:** Result of confirmatory factor analysis of teacher accountability (*n* = 390).

Item	Factor 1	Factor 2
	Internal Accountability	External Accountability
INT1. Be responsible for teaching in the best possible way	0.916	
INT2. Learn from the work of outstanding colleagues	0.909	
INT3. Be ready to use results of studies on instruction and education	0.901	
INT4. Develop professionally in order to accomplish your work in the best way	0.892	
INT5. Be responsible for using professional knowledge in your work	0.880	
INT6. Act by professional ethical principles at your work	0.849	
INT7. Act by your inner moral standards	0.685	
EXT1. Be accountable for your students’ achievements		0.850
EXT2. Be evaluated by whether your students improve their grades		0.839
EXT3. Give school management a report on the extent to which you reached your goals at work		0.815
EXT4. Give yourself a report on the extent to which you reached your goals at work		0.804
EXT5. Pay for the consequences when your work does not meet expectations		0.790
EXT6. Obtain credit for the success of your classes	0.501	0.597

Note. Rotation method: varimax with Kaiser normalization. a rotation converged in three iterations.

**Table 3:** Result of CFA for PAM models.

Model	χ2	χ2/df	RMSEA	NFI	RFI	IFI	TLI
Model 1 * PAM-13a	1373.036	21.124	0.237	0.740	0.688	0.699	0.749
Model 2 ** PAM-13b	325.126	5.502	0.109	0.933	0.918	0.945	0.944
Model 3 * PAM-12c	429.237	8.099	0.141	0.914	0.892	0.904	0.923
Model 3 ** PAM-12c	300.671	5.673	0.111	0.939	0.924	0.949	0.949

Note. * represents subgroup 2 (*n* = 359); ** represents subgroup 3 (*n* = 381); a represents the single-factor, 13-item model; b represents the two-factor, 13-item model; c represents the two-factor, 12-item model.

**Table 4:** Results from reliability, convergent, and discriminant validity analysis.

Model	Factors	Alpha	CR	AVE
Model 2 PAM-13b	Internal accountability	0.960	0.963	0.790
	External accountability	0.911	0.916	0.645

## Data Availability

The data presented in this study are available upon request from the corresponding author. The data are not publicly available due to confidentiality and research ethics.
